# High hydrostatic pressure extract of mulberry leaves ameliorates hypercholesterolemia via modulating hepatic microRNA-33 expression and AMPK activity in high cholesterol diet fed rats

**DOI:** 10.29219/fnr.v65.7587

**Published:** 2021-05-03

**Authors:** Eunyoung Lee, Mak-Soon Lee, Eugene Chang, Chong-Tai Kim, Ae-Jin Choi, In-Hwan Kim, Yangha Kim

**Affiliations:** 1 Department of Nutritional Science and Food Management, Ewha Womans University, Seoul, South Korea; 2 Department of Food and Nutrition, Gangneung-Wonju National University, Gangneung-si, Gangwon-do, South Korea; 3 R&D Center, EastHill Corporation, Gwonseon-gu, Suwon-si, Gyeonggi-do, South Korea; 4 Functional Food & Nutrition Division, National Institute of Agricultural Science (NIAS), Rural Development Administration (RDA), Wanju, jeolabuk-do, South Korea; 5 Department of Integrated Biomedical and Life Sciences, Korea University, Seoul, South Korea; 6 Graduate Program in System Health Science and Engineering, Ewha Womans University, Seoul, South Korea

**Keywords:** adenosine monophosphate-activated protein kinase (AMPK), bile acid, cholesterol, mulberry leaf extract, microRNA-33

## Abstract

**Background:**

Mulberry leaf (*Morus alba* L.) contains multiple bioactive ingredients and has been used in the treatment of obesity, diabetes, inflammation, and atherosclerosis. High hydrostatic pressure (HHP) processing has been developed for the extraction of bioactive compounds from plants. However, the hypocholesterolemic effect of the HHP extract from mulberry leaves and its underlying mechanism have never been investigated.

**Objective:**

The specific aim of the present study was to investigate the hypocholesterolemic property of a novel extract obtained from mulberry leaves under HHP in rats.

**Design:**

Sprague–Dawley rats were divided into four groups and fed either a normal diet (NOR), a high cholesterol diet containing 1% cholesterol and 0.5% cholic acid (HC), an HC diet containing 0.5% mulberry leaf extract (ML), or a 1% mulberry leaf extract (MH) for 4 weeks.

**Results:**

High hydrostatic pressure extract of mulberry leaves significantly reduced the HC-increased serum levels of triglyceride (TG), cholesterol (TC) and low-density lipoprotein cholesterol (LDL-C), and hepatic contents of TG and TC. The HHP extraction from mulberry leaves also increased the HC-decreased fecal TC and bile acid levels without changing body weight, food intake, liver weight, and serum activities of alanine transaminase (ALT) and aspartate transaminase (AST) (*P* < 0.05). The mulberry leaf extract significantly enhanced the expression of hepatic genes such as cholesterol 7 alpha-hydroxylase (CYP7A1), liver X receptor alpha (LXRα), and ATP-binding cassette transporters, ABCG5/ABCG8, involved in hepatic bile acid synthesis and cholesterol efflux (*P* < 0.05). In addition, the HHP extraction of mulberry leaves significantly suppressed hepatic microRNA(miR)-33 expression and increased adenosine monophosphate-activated protein kinase (AMPK) activity.

**Conclusion:**

These results suggest that the HHP extract of mulberry leaves lowers serum cholesterol levels by partially increasing hepatic bile acid synthesis and fecal cholesterol excretion through the modulation of miR-33 expression and AMPK activation in the liver.

## Popular scientific summary

- High hydrostatic pressure extract of mulberry leaves decreased high cholesterol diet-induced serum and hepatic cholesterol levels and increased fecal lipid and bile acid excretion.
- The hypocholesterolemic effect of the mulberry leaf extract appears to be mediated by hepatic mRNA levels related to cholesterol excretion, microRNA-33 expression, and AMPK activity. The mulberry leaf extract has the potential to treat lipid abnormality.

Cardiovascular disease (CVD) has been the leading cause of mortality across the world (1). Hyperlipidemia, which is characterized by increased blood levels of total cholesterol (TC), triglycerides (TG), low-density lipoprotein cholesterol (LDL-C), and their associated lipoproteins, and decreased high-density lipoprotein cholesterol (HDL-C) is considered as a risk factor for CVDs (2). Therefore, it is critical to improve lipid abnormalities to prevent and/or treat CVDs.

Cholesterol metabolism and bile acid synthesis are associated with several transcriptional regulators and enzyme activities. Liver X receptors (LXRs) regulate ATP-binding cassette (ABC) transporters, ABCG5 and ABCG8 expressed in the liver and intestine, thereby controlling cholesterol homeostasis through cholesterol uptake, transport, efflux, and excretion (3, 4). Liver X receptor alpha (LXRα)-deficient mice lack the expression of the rate-limiting enzyme for bile acid synthesis, cholesterol 7 alpha-hydroxylase (CYP7A1), resulting in accumulation of cholesterol in the liver, which leads to CVDs (5). LXRα agonists have been shown to decrease atherosclerosis by mobilizing cholesterol from the periphery, promoting hepatic excretion, and limiting absorption (3, 6). Therefore, cholesterol metabolism and bile acid synthesis might be potential therapeutic targets for dyslipidemia and CVDs.

Mulberry leaf (*Morus alba* L.) is widely used to feed silk worms and as an alternative medicine with anti-obesity, anti-diabetic, anti-inflammation, and anti-atherosclerosis effects (7–11). Growing evidence demonstrates that multiple bioactive components such as alkaloids including 1-doxynojirimycin (1-DNJ), flavonoids, polyphenols, and polysaccharides are found in mulberry leaves which have physiologically beneficial effects (12–14). An aqueous extract of mulberry leaves inhibits the oxidation and lipid peroxidation of LDL in murine macrophage cells (9) and decreases the serum lipid profiles by regulating fatty acid oxidation, lipogenesis, and cholesterol clearance in high fat diet-fed rats or hamsters (15, 16). These studies demonstrate the protective effect of mulberry leaf extract on CVDs. A novel technology, HHP has been developed as an alternative to conventional high-heat processing (17). This non-thermal food processing technology at lower temperatures between –20 and 60°C and pressures above 100 MPa retains sensory and nutritional qualities by disrupting the cell membranes and releasing the intracellular products without thermally degrading the activity and structure of bioactive components (18, 19). In addition, the effectiveness of the mulberry juice’s microbiological quality, bioactive compounds, anti-oxidant activity and volatile profiles was evaluated using HHP technology suggesting that HHP processing might be an alternative to conventional heat treatment for the production of high-quality mulberry juice (20). In the present study, we investigated the hypocholesterolemic effect of HHP-treated mulberry leaf extract in rats fed a high-cholesterol diet. We evaluated several parameters such as lipid levels in serum, liver, and feces, hepatic gene expression related to cholesterol uptake and excretion, microRNA-33 (miR-33) expression, and adenosine monophosphate-activated protein kinase (AMPK) activity.

## Materials and methods

### Preparation of *high hydrostatic pressure*-mulberry leaf extract

The mulberry leaf extract prepared by using the HHP method was kindly supplied from Korea Food Research Institute (Wanju, Jeolabuk-do, Korea). Mulberry leaves were harvested locally (Sangju, Geongsangbuk-do, Korea) in May 2017 and 500 g of the leaves were homogenized in a Waring blender for 5 min. A total of 40,000 units of enzymes of Pectinex ultra color (Daejong Trade Co., Korea) and Pectinex BE XXL (Daejong Trade Co., Korea) were added to the ground mulberry leaves. The mixture was then poured into plastic bags and transferred to a high-pressure apparatus (TFS-50L, Innoway Co. Ltd., Korea), where it was subjected to a pressure of 100 MPa for 4 h at 50 °C. The enzymes were inactivated by boiling at 100 °C for 10 min. The extracts were then cooled and centrifuged at 11,000 *g* for 5 min and filtered through a Whatman No. 5 filter paper. These extracts were then lyophilized and stored at −20 °C until further use.

### Ultra performance liquid chromatography-photodiode detector-quadrupole/time of flight-mass spectrometry (UPLC-PDA-Q/TOF-MS) analysis

Polyphenols in HHP extract of mulberry leaves were identified and quantified using an ACQUITY Ultra Performance LC system. This system was equipped with a photodiode array detector with a binary solvent manager (Waters Corporation, USA) series and a mass detector G2 Q/TOF micro mass spectrometer (Waters, UK) equipped with an electrospray ionization (ESI) source as previously described (21). Individual polyphenols were separated using a Kinetex column (1.7 μm XB-C18 100 A, 150 × 2.1 mm; Phenomenex, USA) at 30 °C. The samples (2 μL) were injected and eluted within 40 min with a sequence of linear gradients. The sample flow rate was set at 0.3 mL/min and the wavelength at 350 nm. The mobile phase comprised solvent A (0.5% formic acid in water, v/v) and solvent B (0.5% formic acid in acetonitrile). The amount of quercetin was calculated by using the following formula:

Content (mg/100 g) = ([*P*1÷*P*2]×*C*×dilution factor)/1,000×100,     (1)

where *P*1 is the peak area of the sample, *P*2 is the peak area of the internal standard, and *C* is the concentration of the internal standard.

### Animals and diets

Six-week old male Sprague–Dawley rats weighing 180–200 g were purchased (Doo Yeol Biotech, Korea). Each rat was housed individually by caging in a controlled environment maintaining a constant temperature (22 ± 2°C), humidity (55 ± 5%), and a 12-h light and dark cycle. After 1 week of acclimatization with free access to water and a normal chow diet (Harlan 2018S rodent diet, United States), 40 Sprague -Dawley rats were randomly divided into four groups (*n* = 10/group) as follows: a normal diet (NOR), a high-cholesterol diet containing 1% cholesterol and 0.5% cholic acid (HC), an HC diet containing 0.5% HHP extract of mulberry leaves (ML), and a 1% HHP extract of mulberry leaves (MH). A commercial chow diet of Harlan 2018S contains 44.2% carbohydrate, 18.6% crude protein, 6.2% fat, 18.2% fiber, and 5.3% ash. The diet compositions for NOR, HC, ML, and MH groups are shown in Table 1. The body weights and food intake amounts were measured twice a week during the 4-week experimental period. Feces were collected on the last three consecutive days of the experiment and stored at –40°C. After 12 h of overnight fasting, the rats were anesthetized with a mixture of Zoletil 50 (Virbac Laboratories, France) and Rompun (Bayer Korea, Seoul Korea). Blood samples were collected by cardiac puncture, separated by centrifugation (2,800 rpm, 20 min, 4°C), and stored at –40°C until further use. Liver and epididymal white adipose tissue (eWAT) were excised, immediately frozen in liquid nitrogen, and stored at –70°C for further analysis. All the experimental procedures were approved by the Institutional Animal Care and Use Committee (IACUC) of the Ewha Womans University (IACUC No. 17-057).

**Table 1:** The composition of experimental diets (g/kg diet)

Component	NOR	HC	ML	MH
Corn starch	150.0	150.0	150.0	150.0
Casein	200.0	200.0	200.0	200.0
Sucrose	500.0	485.0	484.5	484.0
Corn oil	50.0	50.0	50.0	50.0
Cellulose	50.0	50.0	50.0	50.0
Mineral mix^1^	35.0	35.0	35.0	35.0
Vitamin mix^2^	10.0	10.0	10.0	10.0
DL-Methionine	3.0	3.0	3.0	3.0
Choline bitartrate	2.0	2.0	2.0	2.0
Cholesterol	0.0	10.0	10.0	10.0
Cholic acid	0.0	5.0	5.0	5.0
Mulberry leaves extract	0.0	0.0	5.0	10.0
Total	1,000	1,000	1,000	1,000
Total Calorie (kcal)	3,774	3,714	3,712	3,710
Carbohydrates (% as kcal)	67.3	66.8	66.8	66.7
Protein (% as kcal)	19.6	19.6	19.6	19.6
Fat (% as kcal)	11.6	12.1	12.1	12.1

1 AIN-76 Mineral mix (mg/kg diet);

2 AIN-76 Vitamin mix (mg/kg diet).

NOR, normal diet; HC, high-cholesterol diet containing 1% cholesterol and 0.5% cholic acid; ML, HC + 0.5% high hydrostatic pressure mulberry leaf extract; MH, HC + 1% high hydrostatic pressure mulberry leaf extract. This experimental diet was formulated based on the AIN -76 diet composition.

### Serum biochemical markers

Based on the enzymatic colorimetric methods, serum activities of alanine transaminase (ALT) and aspartate transaminase (AST) and serum levels of HDL-C, TC, and TG were measured using commercial kits (Asan pharmaceutical, Korea) in accordance with the manufacturer’s instructions. LDL-C was calculated by using the Friedewald’s formula (22).

LDL-C = TC – HDL-C – (TG / 5)     (2)

### Determination of hepatic and fecal lipids

Lipids from the liver and feces were extracted using the method of Bligh and Dyer with a slight modification as previously described (23). About 0.1 g wet liver tissues were briefly homogenized in 1.5 mL of 0.9% saline and 7.5 mL of methanol:chloroform (2:1, v:v). A total of 2.5 mL of chloroform was added and centrifuged at 3,000 rpm for 20 min. The lower phase was collected by using a Pasteur pipette, moved to a fresh tube, filtered through Whatman No. 6 filter paper, and subsequently dried and weighed. A solution of n-hexane/isopropanol (3/2, v/v) was employed to dissolve the lipid extract. Collected feces were dried in a dry oven (65°C) for 1 day, ground, and weighed. Fecal lipid extraction was carried out in a way similar to the liver lipid extraction. Hepatic and fecal TG and TC levels were analyzed by using the enzymatic colorimetric method using commercial kits as described here.

### Fecal bile acid analysis

Fecal bile acid was extracted following the fecal lipid extraction method as described earlier. Total bile acids (TBAs) in the feces were analyzed using the TBA-test kit (Wako, Japan) according to the manufacturer’s instructions. In these assays, the reacted substrates create colored end products considered to be directly proportional to the concentration of TBA.

### Histological analysis

Dissected liver tissues from the rats were fixed in 10% formalin solution for 24 h, embedded in paraffin, sectioned, and stained with hematoxylin-eosin (H&E). The histology sections were visualized by using a microscope (Olympus, Japan) at 400× magnification.

### Quantitative real-time polymerase chain reaction

Total RNA was isolated from the liver tissues using Ribo Ex (Geneall Biotechnology Co., Ltd., Korea). Complementary DNA (cDNA) was synthesized from 4 μg of isolated RNA using a Moloney Murine Leukemia Virus (M-MLV) reverse transcriptase (Bioneer Co., Korea). An AccuPower 2X Greenstar qPCR MasterMix (-ROX Dye) (Bioneer Co., Korea) and a fluorometric thermal cycler (Corbett Research, Australia) were used for quantitative real-time polymerase chain reaction (qRT-PCR). Primers used for qRT-PCR are described in the supplementary Table 1. β-actin was used as a reference gene for normalization and the results were relatively quantified using the ΔΔCt method (24) and expressed as a fold-difference compared with the HC group.

The miR-33 expression was measured as demonstrated before (25). cDNA was synthesized by using a miRNA cDNA Synthesis Kit with Poly (A) Polymerase Tailing (ABM Inc., Canada) and amplified using the EvaGreen miRNA qPCR Master Mix (ABM Inc.). The miR-33 expression was normalized to U6 snRNA using the 2^−ΔΔCt^ method.

### Adenosine monophosphate-activated protein kinase activity

An AMPK kinase assay kit (MBL Life Science, USA) was used to measure the AMPK activity. The protein levels were determined using a bicinchoninic acid (BCA) protein assay kit (Thermo Scientific, USA). AMPK activity was normalized to the protein concentration and expressed as a fold change compared with the HC group.

### Statistical analysis

All data are expressed as the mean ± standard error of the mean (SEM). Statistical analyses were conducted using the SPSS software (version 23; IBM Corporation, USA). Significant differences among the different groups were determined by a one-way analysis of variance (ANOVA) following Tukey’s multiple comparison tests. A *p*-value less than 0.05 was considered as statistically significant.

## Results

### Composition of high hydrostatic pressure extract of mulberry leaves

**Fig. 1:**
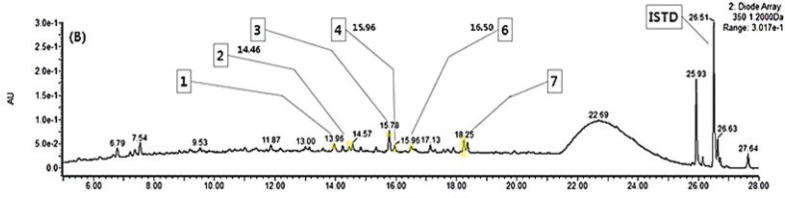
The ultra-performance liquid chromatography (UPLC) chromatogram of flavonoids (detected at 350 nm) in high-pressure mulberry leaf extract. ISTD: internal standard, Peak 1: quercetin 3-O-rutinoside (rutin), Peak 2: quercetin 3-O-glucoside (isoquercitrin), Peak 3: quercetin 3-O-(6’’-O-malonyl)glucoside, Peak 4: kaempferol 3-O-rutinoside (nicotiflorin), Peak 5: quercetin 3-O-(2’’-O-malonyl)glucoside (morkotin), Peak 6: kaempferol 3-O-glucoside (astragalin), Peak 7: kaempferol 3-O-(6’’-O-malonyl)glucoside.

Figure 1 and Table 2 show that the mulberry leaves contain quercetin 3-O-rutinoside (rutin), quercetin 3-O-glucoside (isoquercitrin), quercetin 3-O-(6’’-O-malonyl)glucoside, kaempferol 3-O-rutinoside (nicotiflorin), quercetin 3-O-(2’’-O-malonyl)glucoside (morkotin), kaempferol 3-O-glucoside (astragalin) and kaempferol 3-O-(6’’-O-malonyl)glucoside. Mulberry leaves extracted by HHP contained 14.25 mg phenolic compounds per 100 g of the dried sample.

**Table 2:** Characterization of phenolic compounds of mulberry leaf extract

Compound	mg / 100 g dried sample
Quercetin 3-O-rutinoside (rutin)	2.99 ± 0.33
Quercetin 3-O-glucoside (isoquercitrin)	0.72 ± 0.07
Quercetin 3-O-(6’’-O-malonyl) glucoside	5.00 ± 0.03
Kaempferol 3-O-rutinoside (nicotiflorin)	1.17 ± 0.20
Quercetin 3-O-(2’’-O-malonyl) glucoside (morkotin), Kaempferol 3-O-glucoside (astragalin)	1.79 ± 0.18
Kaempferol 3-O-(6’’-O-malonyl) glucoside	2.58 ± 0.46
Total flavonols	14.25 ± 1.09

Compounds were detected in positive ion mode ([M + H]^+^) using UPLC-PDA-Q/TOF-MS. Each value was calculated as mean ± standard deviation (SD) of three replicates.

### Effects of mulberry leaf extract on body weight, food intake, and adipose tissue mass

After supplementing mulberry leaf extract in the HC diet for 4 weeks, no statistical differences in the final body weight, body weight gain, food intake, energy intake, and energy efficiency ratio were noted for any of the experimental groups. In addition, HHP-treated mulberry leaf extract did not change eWAT mass (Table 3).

**Table 3:** Effect of high hydrostatic pressure mulberry leaf extract on physiological variables

Group	NOR	HC	ML	MH
Initial body weight (g)	216.36 ± 1.28	217.08 ± 1.31	215.59 ± 1.70	215.91 ± 1.40
Final body weight (g)	378.58 ± 5.20	388.87 ± 3.76	380.95 ± 3.35	373.55 ± 5.53
Body weight gain (g / 4 weeks)	162.22 ± 4.59	171.79 ± 3.24	165.37 ± 3.47	157.64 ± 5.33
Food intake (g/day)	23.15 ± 0.47	23.56 ± 0.59	23.25 ± 0.32	23.05 ± 0.65
Energy intake (kcal/day)	87.37 ± 1.78	87.50 ± 2.18	85.87 ± 1.20	84.67 ± 2.38
Energy efficiency ratio (EER)^1^	0.064 ± 0.001	0.068 ± 0.001	0.066 ± 0.001	0.064 ± 0.001
Epididymal fat weight (g / 100 g body weight)	1.52 ± 0.06	1.33 ± 0.04	1.27 ± 0.04	1.36 ± 0.09
Liver weight (g / 100 g body weight)	3.21 ± 0.05^a^	4.75 ± 0.06^b^	4.77 ± 0.07^b^	4.94 ± 0.11^b^
Serum alanine transaminase (IU/L)	13.08 ± 1.98	24.18 ± 3.72	22.32 ± 4.68	20.73 ± 2.93
Serum aspartate transaminase (IU/L)	61.21 ± 7.17	60.84 ± 8.18	63.64 ± 7.93	55.93 ± 6.14

1 Energy efficiency = body weight gain (g/day)/energy intake (kcal/day); Values are expressed as mean ± SEM (*n* = 10).

Different letters (a, b) show significant difference (*P* < 0.05). NOR, normal diet; HC, high-cholesterol diet contained 1% cholesterol and 0.5% cholic acid; ML, HC + 0.5% high hydrostatic pressure mulberry leaf extract; MH, HC + 1% high hydrostatic pressure mulberry leaf extract.

### Influence of high hydrostatic pressure-treated mulberry leaf extract on the liver weight and serum alanine transaminase (ALT) and aspartate transaminase (AST) activities

To investigate whether supplementing the HC diet with the HHP extract from mulberry leaves results in liver toxicity and the HC-induced hepatomegaly, the liver tissue weights, and activities of serum ALT and AST were measured. The HC diet significantly increased the liver tissue weight by about 1.5 fold as compared with the NOR diet, indicating that the HC leads to liver hypertrophy. However, supplementing the HC diet with HHP-treated mulberry leaf extract (ML or MH diet) did not change the liver weight, compared with the HC diet (Table 3). Next, we investigated whether using mulberry leaf extract is safe in rats. Serum ALT and AST activities were not statistically different among the experimental groups.

### Effects of high hydrostatic pressure-treated mulberry leaf extract on serum and hepatic lipid profiles

**Fig. 2:**
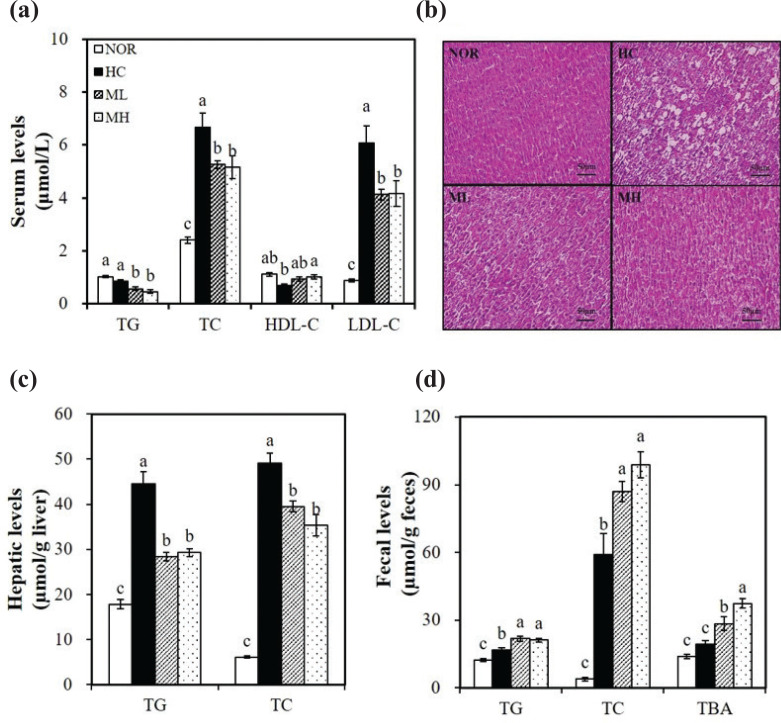
Effects of the high hydrostatic pressure mulberry leaf extract on serum, hepatic and fecal lipid profiles. (a) Serum lipid profiles. LDL-C = TC − HDL-C − (TG/5). (b) Representative hematoxylin and eosin (H&E)-stained liver sections (scale bars, 50 *μ*m; magnification, 400X). (c) Hepatic lipid profiles. (d) Fecal lipid profiles together with bile acid levels. Values are expressed as mean ± SEM (*n* = 10). Bars with different letters (a, b, c) show significant differences (*P* < 0.05). HDL-C, HDL-cholesterol; LDL-C, LDL-cholesterol; TBA, total bile acid; TC, total cholesterol; TG, triglyceride. NOR, normal diet; HC, high-cholesterol diet containing 1% cholesterol and 0.5% cholic acid; ML, HC + 0.5% high hydrostatic pressure mulberry leaf extract; MH, HC + 1% high hydrostatic pressure mulberry leaf extract.

Rats fed on a HC diet had significantly increased serum concentrations of TC and LDL-C and reduced serum concentrations of HDL-C, as compared with rats fed on a NOR diet (*P* < 0.05) (Fig. 2a). The HC-increased serum TC and LDL-C concentrations were significantly reduced by 22.5 and 31.5%, respectively, in the MH group. Supplementing the HC diets with 0.5% or 1% HHP-treated mulberry leaf extract significantly reduced serum TG levels by 33.3% (ML) and 46.3% (MH), respectively (*P <* 0.05) (Fig. 2a). In addition, a 47.9% increase in serum HDL-C concentrations was observed in the MH group (Fig. 2a). After 4 weeks of diet intervention, the HC diet-fed rats showed larger lipid droplets in hepatocytes compared with the NOR diet-fed rats. This feature, which is characterized by fat infiltration in liver tissue, was ameliorated in the mulberry leaf extract-supplemented rats (Fig. 2b). Similarly, hepatic TG and TC levels in HC-fed rats were significantly increased (*P <* 0.05) (Fig. 2c). Supplementation with mulberry leaf extraction significantly reduced the HC-increased hepatic TG and TC levels. This significant decrease in the hepatic TC levels caused by the HHP extract of mulberry leaves was found to be dose-dependent, starting at a dose of 0.5% (*P <* 0.05) (Fig. 2c).

### Effects of high hydrostatic pressure-treated mulberry leaf extract on fecal lipid levels and bile acid excretion

A total of 0.5 and 1% mulberry leaf extract supplementation in the HC diet significantly increased fecal TG levels by at least 1.43 times compared with the HC group (*P* < 0.05) (Fig. 2d). Fecal TC concentrations were significantly increased in a dose-dependent fashion when mulberry leaves were used (*P* < 0.05) (Fig. 2d). In addition, HHP extract of mulberry leaves dose-dependently increased fecal bile acids as measured by TBA levels, reaching statistical significance at the 0.5% dose (ML) (*P* < 0.05) (Fig. 2d). Therefore, it is possible that mulberry leaf extract-reduced serum and hepatic lipid profiles is associated with its-increased fecal cholesterol excretion.

### Effect of high hydrostatic pressure-treated mulberry leaf extract on the expression of hepatic genes related to cholesterol efflux and bile acid synthesis

**Fig. 3:**
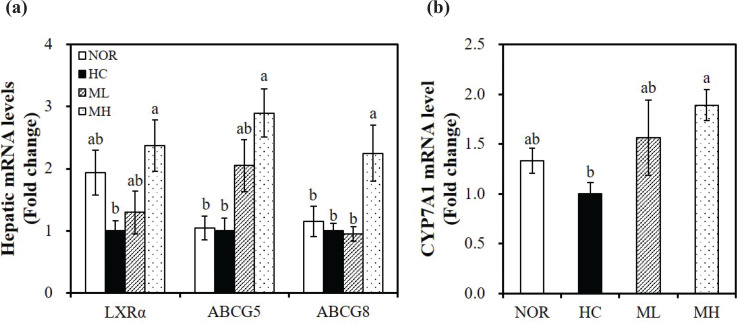
Effects of the high hydrostatic pressure mulberry leaf extract on the expression of hepatic genes related to cholesterol efflux. The mRNA levels of LXRα, ABCG5 and ABCG8 (a), and CYP7A1 (b) were measured by real-time qPCR and normalized to β-actin. The results are expressed as the fold change as compared with the HC group. Values are expressed as mean ± SEM (*n* = 10). Bars with different letters (a, b) show significant difference (*P* < 0.05). NOR, normal diet; HC, high-cholesterol diet containing 1% cholesterol and 0.5% cholic acid; ML, HC + 0.5% high hydrostatic pressure mulberry leaf extract; MH, HC + 1% high hydrostatic pressure mulberry leaf extract.

In the present study, hepatic mRNA levels involved in cholesterol efflux and bile acid synthesis were measured by qRT-PCR. The high hydrostatic pressure extract of mulberry leaves significantly upregulated the HC-decreased hepatic mRNA levels of LXRα, ABCG5, and ABCG8, which are the key transcriptional regulators of cholesterol efflux (*P* < 0.05) (Fig. 3a). Thus, we suggest that the hypocholesterolemic effect of mulberry leaves helps to promote cholesterol efflux through LXRα and ABCG5/8 expression. To investigate the influence of HHP-treated mulberry leaf extract on bile acid synthesis, we measured the hepatic mRNA expression of CYP7A1, a rate-limiting enzyme in bile acid synthesis. Consistent with the increased fecal TBA concentration as indicated by the fecal bile acids, HC-decreased hepatic CYP7A1 gene expression was significantly enhanced by the HHP extraction of mulberry leaves in a dose-dependent manner with increments of 1.56 fold when using ML and 1.89 fold when using MH (*P* < 0.05) (Fig. 3b). Therefore, we suggest that the beneficial effects of mulberry leaves on HC-diet induced hypercholesterolemia may be partially mediated through the regulation of hepatic CYP7A1 expression.

### Effects of high hydrostatic pressure-treated mulberry leaf extract on hepatic miR-33 expression and adenosine monophosphate-activated protein kinase (AMPK) activity

**Fig. 4:**
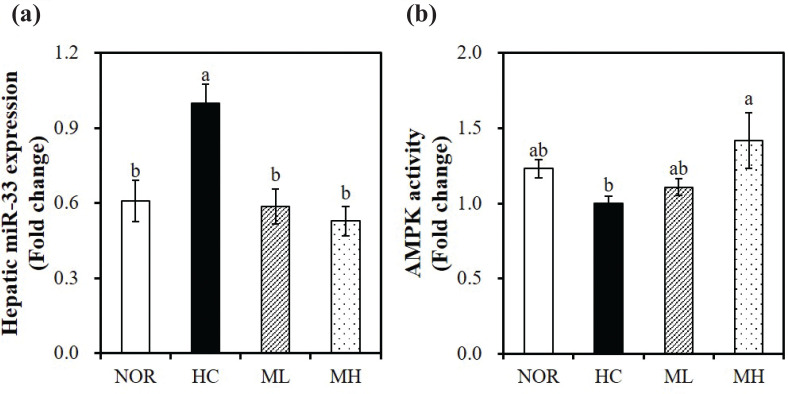
Effects of the high hydrostatic pressure mulberry leaf extract on microRNA (miR)-33 and AMPK activity in the liver. (a) miR-33 expression was analyzed using real-time qPCR, normalized for all samples to U6 snRNA, and expressed as the fold change compared with the HC group. (b) AMPK activity was measured using an AMPK kinase kit, normalized to their relative protein contents, and expressed as the fold change compared with the HC group. Values are expressed as mean ± SEM (*n* = 10/group). Bars with different letters (a, b) show significant difference (*P* < 0.05). NOR, normal diet; HC, high-cholesterol diet contained 1% cholesterol and 0.5% cholic acid; ML, HC + 0.5% high hydrostatic pressure mulberry leaf extract; MH, HC + 1% high hydrostatic pressure mulberry leaf extract.

To investigate the molecular mechanisms by which HHP extract of mulberry leaves regulate cholesterol homeostasis, we measured the hepatic expression of miR-33, a potent post-transcriptional gene regulator in cholesterol efflux, high-density lipoprotein biogenesis, and fatty acid oxidation. In the present study, miR-33 expression in rats fed with HC diet including 0.5 or 1% mulberry leaf extract was significantly downregulated by 41.3 and 47.1%, respectively, compared with HC-fed rats (*P* < 0.05) (Fig. 4a). Next, we analyzed AMPK activity, a cellular energy metabolic sensor involved in the hepatic lipid metabolism at the transcriptional and post-transcriptional levels by regulating fatty acid oxidation and cholesterol and TG synthesis. Supplementing the HC-diet with HHP-treated mulberry leaf extract significantly increased the hepatic AMPK activity in a dose-dependent manner, with a 1.11 fold-increment by 0.5% mulberry leaf-contained HC diet, as compared with the HC diet (*P* < 0.05) (Fig. 4b).

## Discussion

Based on numerous epidemiological studies, hypercholesterolemia has been considered as an important risk factor for CVDs (1, 2, 26). Cholesterol homeostasis is fairly well-regulated in the body, which involves the synthesis of bile acid, the conversion of cholesterol to bile acids in the liver and cholesterol efflux, and biliary cholesterol excretion to feces (27). Natural foods and their bioactive functional ingredients have gained attention due to their multiple pharmacological effects. High hydrostatic pressure has been introduced as a non-thermal food processing technique to keep the activity and structure of bioactive ingredients in foods (18). In the present study, we investigated the favorable effect of the HHP-treated mulberry leaf extract on cholesterol metabolism in rats fed a high-cholesterol diet.

Among multiple bioactive components including 1-DNJ, flavonols, polyphenols, and polysaccharides (12, 14), a previous study detected quercetin 3-glucoside (isoquercitrin) and kaempferol 3-glucoside (astragalin) as flavonols in mulberry leaves (28). High hydrostatic pressure extraction of mulberry leaves contained 14.25 mg total flavonols including isoquercitrin and astragalin in 100 g of the dried sample. In the present study, we evaluated the influence of HHP-treated dietary mulberry leaves on hypercholesterolemia in rats fed with a HC diet. With regard to used two doses determined as previously described (8, 15), no significant differences in serum ALT and AST activities and liver tissue weight were observed. It suggests that both doses of mulberry leaves used in the present study at least did not induce liver toxicity. Consistent with previous studies using hot water extract (15, 16), the HHP extract of mulberry leaves significantly reduced HC-increased serum TC, TG, and LDL-levels. In addition, a dose-dependent increase in serum HDL-C level was observed in rats fed on the HC diet supplemented with the HHP extract from mulberry leaves. Several randomized clinical trials have shown the beneficial effects of mulberry leaves on serum TC, TG, and LDL concentrations in subjects with dyslipidemia without severe adverse reactions (29–31). It suggests that mulberry leaves might be a potential therapeutic resource for modulating hypercholesterolemia and CVD risk.

To investigate the mechanism underlying the cholesterol lowering effect of HHP-treated mulberry leaf extract, we measured the expression of hepatic genes related to cholesterol homeostasis. Cholesterol homeostasis is maintained through the balance between hepatic cholesterol biosynthesis and hepatic cholesterol catabolism (32). In the maintenance of cholesterol homeostasis, bile acids are the end products of cholesterol catabolism, and their biosynthesis and excretion to feces are involved in a reduction of excess hepatic cholesterol (33). In this study, supplementation with HHP-treated mulberry leaves in the HC diet significantly decreased hepatic TG and TC levels and increased fecal TG, TC, and bile acid contents, when compared with the HC diet. In addition, rats that were fed a HC diet with the HHP extract of mulberry leaf extract had higher hepatic LXRα and ABCG5/8 gene expression involved in cholesterol efflux and CYP7A1 mRNA levels related to bile acid synthesis. LXRα regulates its target genes such as ABCA1, ABCA1, ABCG5, ABCG8, and CYP7A1, thereby coordinating the balance between cholesterol biosynthesis and catabolism (3, 4). When cholesterol levels are high, LXRα activation upregulates hepatic and intestinal ABCG5/8 expression (3). Genetic modifications of ABCG5 and ABCG8 in a rodent model depict their role in cholesterol absorption and biliary cholesterol secretion (34, 35). In addition, LXRα upregulates CYP7A1 expression, the rate-limiting enzyme in bile acid synthesis and cholesterol excretion (5). It suggests that the hypocholesterolemic effect of HHP-treated mulberry leaf extract might at least, in part, interact with LXRα nuclear receptor and its-target genes, ABCG5/8 and CYP7A1 in the liver.

Apart from the classical transcriptional regulators, miRNAs, members of a class of non-coding RNAs have been identified as critical post-transcriptional regulators of cholesterol homeostasis by binding to complementary target sites in the 3’ untranslated regions of mRNAs (36). The miR-33 expression has been shown to post-transcriptionally inhibit key target genes such as ABCA1 and ABCG1 involved in cholesterol efflux and high-density lipoprotein biogenesis, and fatty acid oxidation-related genes including carnitine palmitoyltransferase 1α (CPT1α), carnitine O-octanoyltransferase (CROT), hydroxyacyl-CoA dehydrogenase/3-ketoacyl-CoA thiolase/enoyl-CoA hydratase beta subunit (HADHB), and AMPK (37). During increasing fatty acid oxidation, AMPK, a critical energy sensor and metabolic master switch, inhibits hepatic cholesterol and TG synthesis (38). Previous studies show that mulberry leaf extract or mulberry leaf polyphenol extract activates AMPK phosphorylation and inhibits hepatic lipogenesis (39, 40). In addition, AMPK activation suppresses endogenous LXR ligand production leading to the decrease in LXR expression and its transcriptional regulation (41). In this study, the HHP extract of mulberry leaves significantly decreased HC-induced miR-33 expression and increased HC-decreased AMPK activity in the liver. Taken together, mulberry leaf extract-mediated modulation of miR-33 expression and AMPK activity might be essential in regulating cholesterol homeostasis by increasing bile acid synthesis and cholesterol efflux.

## Conclusion

This study has demonstrated for the first time that a 4-week supplementation with HHP extraction of mulberry leaves improves the HC diet-induced serum and hepatic lipid abnormalities and increases fecal lipid excretion in rats fed a high cholesterol diet. The mulberry leaf extract also increases fecal bile acid content, which is accompanied by an increase in the expression of hepatic genes involved in cholesterol metabolism, miR-33 expression, and AMPK activity. These findings suggest that HHP-treated mulberry leaf extract has the potential to prevent/treat hypercholesterolemia and CVDs associated with it.

## Supplementary Material

High hydrostatic pressure extract of mulberry leaves ameliorates hypercholesterolemia via modulating hepatic microRNA-33 expression and AMPK activity in high cholesterol diet fed rats
