# Supplementary material for: High hydrostatic pressure extract of mulberry leaves ameliorates hypercholesterolemia via modulating hepatic microRNA-33 expression and AMPK activity in high cholesterol diet fed rats
Source: Food Nutr Res. 2021 May 3;65:10.29219/fnr.v65.7587. doi: 10.29219/fnr.v65.7587 (PMC8098647; doi:10.29219/fnr.v65.7587)
Supplement: High hydrostatic pressure extract of mulberry leaves ameliorates hypercholesterolemia via modulating hepatic microRNA-33 expression and AMPK activity in high cholesterol diet fed rats [file FNR-65-7587-s001.docx]

***Supplementary Table 1.*** Primers used for quantitative real-time PCR

| Name | Genbank No. | Primer sequence (5’-3’) | Amplicon size (bp) |
| --- | --- | --- | --- |
| β-actin | NM_053365 | F: GGCACCACACTTTCTACAAT  R: AGGTCTCAAACATGATCTGG | 123 |
| LXRα | NM_031627 | F: GACTTCGAGTCACGCCTTGG  R: GTCCTCCCTGCTCAGCTGTA | 161 |
| ABCG5 | NM_053754 | F: ATGAGTGAGCTGCCCTTTCT  R: CGCTGAAGGACACATTCAGG | 142 |
| ABCG8 | NM_130414 | F: ACCCTCACACAGGACACCAA  R: GGATGAACAGGGTGGGCAAG | 124 |
| CYP7A1 | NM_012942 | F: TGTTCTGTGTTCACTTTCTG  R: ACTCGGTAACAGAAGGCATA | 126 |

ABCG5, ATP-binding cassette sub-family G member 5; ABCG8, ATP-binding cassette sub-family G member 8; CYP7A1, Cholesterol 7 alpha-hydroxylase; LXRα, liver X receptor α
